# An evaluation of outcomes, interventions and behaviour modifications following a cardiovascular MRI in HIV infected patients

**DOI:** 10.1186/1753-6561-9-S1-A30

**Published:** 2015-01-14

**Authors:** N Aftab, C Doherty

**Affiliations:** 1 Trinity College Dublin and The Guide Clinic, St. James Hospital, Dublin 8. Ireland

## Background

The advent of antiretroviral therapy has led to increased life span which has been associated with an increase in cardiovascular risk factors and heart disease (Boccara F et al, 2013 [1]). Cardiovascular risk assessment is now advised for those with HIV. The field of behavioural cardiology is particularly applicable in HIV therapy due to the long term nature of such a condition. Therefore, it’s useful to assess behaviour modifications following comprehensive cardiac evaluation, specifically CMRI.

## Methods

This is a cross-sectional study examining the medical outcomes, interventions and behaviour modifications in a cohort of 184 male (169 HIV positive compared to 21 HIV negative controls), HIV positive patients at the GUIDE Clinic. The purpose of the study was to compare these variables in 148 patients who underwent a cardiac MRI (CMRI) versus 36 patients who underwent a Framingham risk assessment. The initial study involved the identification of cardiac abnormalities by blood tests and CMRI scan, while the follow-up involved changes in lifestyle, medications as well as cardiac relevant events.

## Results

When comparing the two groups, 8.8% of the CMRI group quit smoking while only 2.8% of the Framingham group did. Of the CMRI group, 7.4% commenced new cardiac medication while commencement in the Framingham group was 8.3%. Of the CMRI group 16.2% had further cardiac investigations after their CMRI compared to 8.3% in the Framingham group.

## Conclusions

Having a cardiac MRI seems to be more effective in producing behavioural modifications as well as more precise medical diagnoses in HIV positive patients. Ideally, Framingham Risk Assessments should be altered to include HIV specific CVD risk factors; however this model does not yet exist. Due to the increased CVD risk factors seen in HIV positive patients, cardiac assessments need to be performed more routinely with a lower threshold for further cardiac investigations.
